# Me, Too

**DOI:** 10.1200/JGO.2015.000588

**Published:** 2016-01-20

**Authors:** Bishal Gyawali

**Affiliations:** Department of Hemato-Oncology, Nobel Hospital, Sinamangal, Kathmandu, Nepal.

Every year at the annual ASCO meeting, the economic unfeasibility of anticancer therapies becomes a hot topic of discussion. The skyrocketing cost of newer therapies, especially the targeted drugs and immunotherapeutic agents, has taken cancer treatment beyond the reach of the majority. At the ASCO Annual Meeting in 2015, a speech at the plenary session by Dr Leonard Saltz on the cost of anticancer therapies provoked a big discussion, both in the oncology community and the media.^1^

Some points raised by Dr Saltz are particularly noteworthy. That the median monthly price for new anticancer drugs has risen from $4,716 in 2000 to 2004 to $9,900 in 2010 to 2014 is staggering. The fact that there are no regulations on pricing of new anticancer drugs is both absurd and sad. That a pharmaceutical company is free to price its drug at whatever price it deems appropriate, on the basis of what the market will bear,^2^ and that the US Government, via Medicare, is obligated to purchase the drug at that price and is barred by law from negotiating price, is illogical, baffling, and inhumane. The affordability of cancer treatment cannot be treated similarly to that of commodities such as cars or paintings.^3,4^

Dr Saltz commented on a study presented earlier in that same plenary session, which showed that the combination of nivolumab and ipilimumab resulted in a median progression-free survival of nearly 1 year in patients with metastatic melanoma,^5^ but at a cost of nearly $295,000 per year per patient just for those two drugs. In 2012, of the 13 new drugs approved for cancer, 12 were priced at more than $100,000 per year.^6^ Yes, progress comes at a price, but there is a limit to what we, as a society, can afford.

As spokespersons for the pharmaceutical industry often point out, the research required to develop new drugs requires investment and risk, with many agents ultimately not making it to market. However, the industries’ estimates of drug development costs are often highly inflated and rarely, if ever, supported by an open sharing of corroborating data.^2,3^ The disconnect between development costs and drug price is exemplified by the price of imatinib, which started at $30,000 per year in 2001 but increased to $92,000 per year in 2012, despite there being no additional development costs during that time. Further, the price did not drop but rather rose when newer competing agents, such as dasatinib and ponatinib, came on the market. In addition, as new indications for imatinib were added, making the potential market larger, the price continued to increase, further demonstrating the disconnect between drug pricing and development and production costs.^3,7,8^

Recently, there was a Comments and Controversies article published in *Journal of Clinical Oncology* regarding the inequalities in approval of the same drug across different countries.^9^ The authors expressed their concern that bevacizumab, though approved in the United States and Europe for epithelial ovarian cancer, is not approved in Canada or the United Kingdom. However, the issue I want to highlight is that not only bevacizumab but nearly all anticancer drugs, both old and new, are out of reach for people living in low- and low-middle–income countries (LLMICs). I represent Nepal, a small country sandwiched between India and China, which was poor even before the recent devastating earthquake. While oncologists at the ASCO meeting were debating the value and affordability of new targeted drugs and immunotherapies, patients in my country were deciding whether to sell their house for the treatment of their mother’s ovarian cancer with paclitaxel and carboplatin, or to accept best supportive care as first-line treatment. There would be no point in talking about bevacizumab; it would be out of the realm of consideration. The affordability of generic paclitaxel and carboplatin is what would be in question.

In 2014, a Lancet Oncology Commission highlighted the status of cancer epidemiology, treatment, and research in the developing countries of India, China, and Russia.^10^ These countries constitute one-third of the world’s population and one-half of the total cancer burden, with a mortality rate nearly twice that of the United States or the United Kingdom.^10^ But these countries are sidelined when it comes to accessibility of cancer therapies. When countries as large as India and China receive insufficient attention, it is not at all a surprise that a small country sandwiched between them is completely forgotten. GLOBOCAN estimates that more than 65% of cancer deaths occur in low-income countries (LICs),^11^ although only 5% of global cancer-care resources are directed toward these countries.^12^

Nepal shares many similarities with India, including religions, culture, values, and languages, as well as the lack of a national health insurance scheme. This 100% pay-from-the-pocket model severely cripples oncologists from providing even the most basic services to patients, most of whom live well below the poverty line. The government of Nepal has allocated an allowance of 50,000 rupees (approximately US$500) for each cancer patient, which is not enough to purchase even one vial of trastuzumab.^13^ Countries like Nepal and India need a different model of national insurance, in concordance with the local economic capacity and feasibility.^13^ However, even if an insurance system were to come into place, the government of Nepal, and even that of India, could never bear the current high costs of newer anticancer therapies.

Despite such difficult circumstances, Nepal had been slowly trying to raise its oncology standards with the popularization of outpatient chemotherapy, as well as beginning bone marrow transplant services in 2012.^13a^ However, the aftermath of the earthquake is sure to divert the attention of the government to rebuilding and to the most basic health care. Concerns are already arising regarding vector-borne communicable diseases and maternal-child care in the postearthquake era.^14,15^ No article, however, has yet raised the question of cancer care in postearthquake Nepal. This lack of attention, as well as other high-priority issues after the earthquake, will further limit the oncology services in this small nation, already crippled by lack of resources, difficult geography, and a severe lack of education. It should be remembered that we are discussing providing cancer-care access to populations who truly believe that the earthquakes are due to our mountains getting angry.^16^

How to incorporate the newer agents in the United States or European health systems should not be the only priority; how to make these agents affordable even in LLMICs like Nepal should also be a matter of discussion. There is no denying that the accessibility of expensive cancer drugs to all is still a problem in countries like the United States^17^; however, the oncology community cannot and should not move forward ignoring half of the patients with cancer in the world. How or why should anyone be excited about these advances if nearly half of those who need them are not going to receive them because of cost?

Lack of educated manpower has been identified as an important challenge for cancer control in developing nations.^10^ However, I am at a loss as to what the education of oncologists in Nepal should constitute. Being well versed in the major cancer treatment guidelines is more of a pain than solace in such a resource-limited setting. While the developed world is worried about the economic viability of incorporating sipuleucel-T, we oncologists in the South Asian Association for Regional Cooperation region are worried about the economic feasibility of incorporating generic docetaxel into the management of prostate cancer. Pertuzumab and trastuzumab emtansine are not the concerns in our world, where people are not able to afford even trastuzumab. For the patients and oncologists of Nepal, the finding that palbociclib is effective in breast cancer^10^ is similar to the discovery of black holes: quite exciting from a scientific point of view, pointless from practical point of view. Under such circumstances, practice of evidence-based medicine seems a far-fetched dream. If oncologists in Canada feel sad about not being able to prescribe bevacizumab,^3^ consider how it must feel to be unable to prescribe nearly 100% of targeted and immune therapies because of unaffordability! The era of targeted therapies has not yet dawned in half of the world. I, although in Japan now, get excited about every new drug discovery in cancers. But when I am back home in Nepal, such news brings despair because that is one more drug that I could use, if only we could afford it.

So how can we enable poor patients from LLMICs to benefit from progress in cancer treatment? One way is to bring awareness and attention to the problem. The recent earthquake serves as a good metaphor for cancer control in LLMICs. The media did an excellent job of covering the natural catastrophe, which brought the national and international authorities and aid agencies together to deal with this disaster. Lack of access to anticancer treatment is also a catastrophe but on an international scale involving more people.^4^ Oncologists must bring this issue forward and encourage the media and responsible authorities to take notice. Other approaches need to focus on the costs of cancer care and what can be done to mitigate them. The high costs of cancer care in the developed world serve as the starting point from where prices in the undeveloped world are established.

Tabernero^18^ recently highlighted that there are too many “me too” drugs being developed in oncology. Developing many drugs belonging to the same category with little or no difference adds to development costs but adds little benefit. Although we can hope that such agents will stimulate competition and drive down prices, we have yet to see evidence of this.

Another opportunity that has yet to be explored and could have important positive global economic impact is early-phase clinical trials conducted in countries like Nepal and India, as we have previously proposed.^8^ Because the cost of supportive management is low compared with that in other nations, and the health system in these countries is English-language based, conducting phase I and II trials in these nations is much cheaper.^8^ This helps reduce the cost of cancer-drug development globally and also allows earlier access to these new drugs for the poor patients—a win-win. There certainly are some peculiar challenges to conducting clinical trials in LLMICs, such as overly enthusiastic endeavors from both the patients and the oncologists for inclusion in the trial, because the alternative is bleak best supportive care as a result of unaffordability of the standard treatment. However, such challenges could be addressed by rigorous monitoring of the trials and education of the oncologists and patients. One important but frequently neglected strategy is conducting research to explore cheaper alternatives to expensive therapies. Finding a cheaper alternative to an expensive therapy should be considered as important as finding a new drug. The impact on global health of the discovery of visual inspection with acetic acid to screen for cervical cancer is more significant than discovering ramucirumab’s efficacy in gastric cancer, because 85% of cervical cancers occur in low income countries (LICs)^11^ and these results from India are directly translatable to Nepal and other LICs.^19,20^ One more example is the recent publication from India of elective versus therapeutic neck dissection in oral cancers.^21^ Although it may not translate directly to high-income countries because the observation cohort underwent clinical palpation instead of imaging studies during follow-up, it is of high importance to countries like Nepal. The presentation of the paper by D’Cruz in the plenary session of ASCO and its subsequent publication in the *New England Journal of Medicine* represents a pleasant paradigm-shifting acceptance of the global community to the need and importance of research in LLMICs.

We recently reported that a cheaper version of amphotericin B emulsified in lipids could be used as a substitute for expensive liposomal amphotericin for antifungal prophylaxis during induction chemotherapy in patients with leukemia in Nepal.^9^ Research done in countries like Nepal, exploring the cost-effective approaches to treatment, can be easily translated to the West.^22^ The recommendations made by the International Agency for Research on Cancer regarding the futility of ultrasonography, mammography with tomosynthesis and magnetic resonance imaging in breast cancer screening also present an important cost-saving finding,^23^ given that breast cancer is the leading cause of death in LICs.^24^

LLMICs provide a unique opportunity for cancer research because certain cancers (e.g., virus-associated cancers) have a higher incidence in these regions. An active collaboration between researchers from developed countries and LLMICs could help elucidate unique strategies to combat these challenges.^25^ LLMICs like Nepal also provide a unique opportunity to conduct clinical trials on cheaper options of supportive care, such as olanzapine for chemotherapy-induced nausea and vomiting, that are unlikely to be industry sponsored and conducted in the developed world.^26^ Olanzapine is a commonly used antipsychotic easily and cheaply available even in countries like Nepal and has already shown promising results in trials of chemotherapy-induced nausea and vomiting.^27,28^

Oncologists themselves cannot completely solve these problems. Nevertheless, oncologists can indeed play a pivotal role if they are organized and united. Although oncologists cannot change rules and make policies, we are not entirely powerless. Just as an example, the price of ziv-aflibercept was reduced by half within 1 week of publication of an editorial in *The New York Times* that highlighted the ridiculous expensiveness of ziv-aflibercept compared with bevacizumab despite similar benefits in colorectal cancer.^29^ Similar to the control of profiteering by the government of Nepal after the earthquake, oncologists should join hands to protest the inflated pricing of drugs by the companies, because cancer is also a natural disaster and excessive pricing of drugs is profiteering to that end.^3^

We must also promote research not just on new drug development but also on the cost efficacy of available drugs; for example, the economic analysis of adding ixabepilone to capecitabine^30^ or the economic analysis of CALGB/SWOG 80405.^31^ In fact, the journals should require all the phase III positive trials to report economic analyses as a part of their publication, similar to reporting of adverse effects. Financial burden should also be acknowledged as an important adverse event, not just for the patient but the whole family, country, and humanity.

Some common cost-saving measures oncologists all over the world can individually accomplish include avoiding tumor-marker surveillance, which has no benefit in most cancers, stopping the use of chemotherapy for patients with a performance status of greater than 2, and curbing the rampant use of granulocyte-colony stimulating factors despite the lack of evidence of meaningful benefit.^32^ Another area in which we oncologists can change is our own behaviors. It has been demonstrated that more generously reimbursed physicians tend to prescribe more expensive regimens.^33^

ASCO has slowly but surely been expanding its network of responsibility across the globe. Along with the frequent International Clinical Trial Workshops they hold in developing countries (there was one in Nepal in 2014), ASCO has also now launched a new journal to particularly address the developing world, *Journal of Global Oncology*. Furthermore, I am hopeful that ASCO’s first ever clinical trial, Targeted Agent and Profiling Utilization Registry (TAPUR) study, will be a blessing to many poor patients who would otherwise never have access to these drugs. ASCO has also recently published a framework to assess the value of cancer treatment options.^34^ This is a helpful strategy to make treatment decisions in both developed and developing countries, although further work is needed to make it more easily and swiftly usable in busy clinics. Such academic organizations also have a role to motivate the industry to fund research in LLMICs and pressure the companies on their corporate social responsibilities.^35^ It is no wonder that a recent survey among oncologists showed that oncologists from high-income countries were more involved in industry-sponsored research than those from LICs, and oncologists from LICs cited lack of funding as the most important obstacle.^36^ The Breast Health Global Initiative is another example of how oncologists can work together to make some difference.^37,38^ After the Breast Health Global Initiative, the National Comprehensive Cancer Network panel has also recently announced the launch of its guidelines on the basis of economic feasibility; the cervical cancer guidelines are already in place.^39^ Similarly, the International Federation of Gynecology and Obstetrics staging system for gynecologic cancers does not involve expensive imaging and yet can help inform important decisions.^40^

In conclusion, the underdeveloped world cannot be ignored in our planning of the global combat against cancer. The fundamental step is acknowledging the problem. I have found a number of editorials and perspective pieces in highly reputed journals on the issue of economic feasibility of anticancer treatment, but all of them focused on developed countries alone, ignoring more than half of the global cancer burden, which occurs in LLMICs.^3,4,6,29,32,41-46^ Acknowledgment of the global problem is the first step. As a representative of the oncologists and our patients from the underdeveloped world, I am saying “Me, too.”
